# New vessels formation in young strokes with isolated steno‐occlusive MCA

**DOI:** 10.1002/brb3.1088

**Published:** 2018-09-15

**Authors:** Meng Liang, Peng Wang, Yan Ma, Xiaohao Zhang, Jie Gao, Minmin Ma, Zhengze Dai, Dezhi Liu, Xinfeng Liu

**Affiliations:** ^1^ Department of Neurology Jinling Hospital Second Military Medical University Nanjing Jiangsu China; ^2^ Department of Medical Imaging Jinling Hospital Second Military Medical University Nanjing Jiangsu China; ^3^ Department of Neurology Second Afliated Hospital of Nanjing University of Chinese Medicine Nanjing Jiangsu China; ^4^ Department of Neurology Jinling Clinical College of Nanjing Medical University Nanjing Jiangsu China; ^5^ Department of Neurology Shanghai General Hospital Shanghai Jiao Tong University School of Medicine Shanghai China

**Keywords:** collaterals, ischemic stroke, MCA, young

## Abstract

**Objective:**

New vessels formation (NVF) along the steno‐occlusive middle cerebral artery (MCA) is often observed in digital subtraction angiography (DSA) imaging. In the study, we aim to explore the clinical relevance of NVF in young ischemic stroke patients with isolated middle cerebral artery (MCA) stenosis.

**Methods:**

We retrospectively reviewed the digital subtraction angiography (DSA) images of 93 young ischemic stroke patients (age ≤ 45 years old) in our center from January 2006 to June 2016. All the patients were diagnosed with isolated steno‐occlusive middle cerebral artery (MCA) disease.NVF was defined as new vessels formation along the stenotic MCA on anteroposterior DSA projection. The association between NVF and functional outcome was analyzed.

**Results:**

The prevalence of NVF was 0 in moderate stenosis, 15.8% in severe stenosis, and 53.7% in MCA occlusions. The presence of NVF had a strong correlation with the severity of MCA stenosis (*r *=* *0.467, *p *<* *0.001). Compared to patients without NVF, patients with NVF were more likely to suffer an unfavorable functional outcome (6.2% vs. 21.4%, *p* = 0.061) at 3 months. Univariate logistic regression analysis showed that NVF was associated with unfavorable outcome [Odds Ratio (OR) = 4.159, 95% confidence intervals (CI) = (1.072, 16.137), *p *=* *0.039].

**Conclusions:**

This study demonstrated that NVF were associated with poor clinical outcome in young ischemic stroke patients who were diagnosed with isolated steno‐occlusive MCA.

## 1 INTRODUCTION

For patients with ischemic stroke, young adults (age ≤ 45 years old) account for 10 percent of overall incidence, and the proportion is still increasing (George, Tong, & Bowman, 2017; Maaijwee, Rutten‐Jacobs, Schaapsmeerders, van Dijk, & de Leeuw, 2014). Middle cerebral artery steno‐occlusive disease (MCAD) is a common etiology for ischemic stroke in young adults (Ahn et al., 2015; Huang et al., 1997; Wong et al., 1998). Under some pathological conditions, new vessels formation (NVF) can compensate for distal hypoperfusion induced by MCA stenosis or occlusion (Sheth & Liebeskind, 2015). Due to the difference of collateral circulation status, patients with MCAD may present with different symptom severity, functional outcome, and rate of recurrence (Liebeskind, Cotsonis, Saver, Lynn, & Cloft, 2011). This study aimed to explore the clinical relevance of NVF in young ischemic stroke patients who were diagnosed with isolated steno‐occlusive MCA by digital subtraction angiography (DSA) examination.

## 2 MATERIALS AND METHODS

### 2.1 Patients

We retrospectively screened the patients of Nanjing stroke register program between June 2006 and October 2016. Patients (age ≤ 45 years old) with symptomatic isolated MCA stenosis (≥50%) or occlusion were included in the current study. All the patients were diagnosed with ischemic stroke by MRI or CT scans.

The exclusion criterions were as follows: (a) bilateral MCA abnormalities such as definite moyamoya disease, (b) ipsilateral stenosis or occlusion involved the terminal portion of internal carotid artery (ICA) or the proximal area of anterior cerebral artery (ACA), (c) coexisting ≥50% other cerebral artery stenosis, (d) evidence of cardioembolism confirmed by ECG, UCG or Holter monitoring, (e) vasculitis or arterial dissection confirmed by clinical information, laboratory data and imaging results; (f) time from symptom onset to DSA examination <30 days. The present study was approved by the institutional review board of our hospital,and informed consent was obtained from all participants or their relatives.

### 2.2 Angiographic assessment

**Figure 1:**
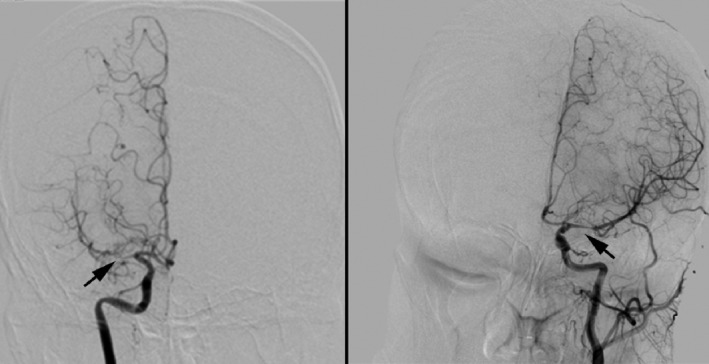
Two patients with severe isolated MCA stenosis, NVF was found in the left patient and no NVF was found in the right patient (showed by the black arrow). MCA: middle cerebral artery; NVF: new vessels formation. MCA: middle cerebral artery; NVF: new vessels formation

All patients underwent DSA examination for etiological diagnosis, which were performed with an AXIOMARTIS dTAAngio Lab (SIEMENS Medical System, Germany). Two experienced neuroradiologist reviewed the DSA images and differences were resolved by consensus. They were both blinded to the clinical information of the patients. NVF was defined as new vessels formation along the affected MCA on anteroposterior DSA projection (Figure 1). The degree of MCA stenosis were calculated by the formulation: degree of stenosis = 1–*D*_stenosis_/*D*_normal_ × 100% (Samuels, Joseph, Lynn, Smith, & Chimowitz, 2000). The degree of MCA stenosis was classified into 3 categories: moderate (50% ~ 70%), severe (70% ~ 99%), and occlusion (100%). The status of collateral circulation was assessed using American Society of Interventional and Therapeutic Neuroradiology/Society of Interventional Radiology (ASITN/SIR) Collateral Flow Grading System. The categories are as follows: 0 = no collaterals visible to the ischemic site; 1 = slow collaterals to the periphery of the ischemic site with persistence of some of the defect; 2 = rapid collaterals to periphery of ischemic site with persistence of some of the defect and to only a portion of the ischemic territory; 3 = collaterals with slow but complete angiographic blood flow of the ischemic bed by the late venous phase; 4 = complete and rapid collateral blood flow to the vascular bed in the entire ischemic territory by retrograde perfusion (Higashida & Furlan, 2003). Then, grades 0, 1, 2 were categorized as poor status of primary and secondary collateral circulation and 3, 4 were categorized as good status of primary and secondary collateral circulation. The inter‐observer consistency for NVF and collateral circulation status was tested in random selected 10 cases, which were both excellent (*κ *= 0.85, *κ* = 0.90).

### 2.3 Clinical assessment

The clinical data including demographic information, stroke risk factors, and laboratory data were collected with a standard case report form. Stroke severity of the patients was assessed by National Institutes of Health stroke scale (NIHSS) (Brott et al., 1989). The information of functional outcome and recurrence was obtained through regular outpatient follow‐up or structured telephone interview. The modified Rankin scale (mRS) was used to evaluate the functional outcome at 3 months. Functional outcome was defined as favorable (mRS0‐2) and unfavorable (mRS3‐6). Recurrence was defined as a new neurological defect in the same MCA territory after the first stroke. According to clinical diagnostic criteria, recurrence was clarified into stroke and TIA.

### 2.4 Statistical analysis

Statistical analysis was performed with SPSS software version 24.0 (http://scicrunch.org/resolver/SCR_002865, IBM). Continuous variables were described as mean (*SD*) or median (interquartile range, IQR), and analyzed with a *t* test or Mann‐Whitney *U* test. Categorical variables were expressed as *n %* and compared by chi‐square test or Fisher's exact test. Spearman's correlation analysis was performed to evaluate the correlation between severity of stenosis and prevalence of NVF. Logistic regression analysis was performed to study the association between NVF and functional outcome. A 2‐tailed *p* value less than 0.05 was considered statistically significant.

## 3 RESULTS

According to the inclusion and exclusion criterions, 93 patients with an average age of 37.9 ± 5.5 years (from 23 to 45 years old) were included in our study. Males were predominant in our cohort (83.9%).The median NIHSS score at admission was 3 (0, 7.5). After evaluating the DSA images, NVF was found in 28 (30.1%) patients. The prevalence of NVF was 0 in moderate stenosis, 15.8% in severe stenosis, and 53.7% in MCA occlusions. The presence of NVF had a strong correlation with the severity of MCA stenosis (*r *=* *0.467, *p *<* *0.001) (Table 1).There was a trend that NVF was more prevalent in patients with poor status of primary and secondary collateral circulation (*p *=* *0.055). Among patients with or without NVF, there were no significant differences in demographic information or stroke risk factors. There was a trend that patients with NVF tend to have an unfavorable outcome than patients without NVF at 3 months (*p *=* *0.061). At a mean 53.7 months (range 6 to 120 months) of follow‐up, 5 (18.9%) patients with NVF, as well as 7 (10.9%) patients without NVF suffered recurrent events (*p *=* *0.330) (Table 2). Univariate logistic regression analysis showed that NVF was associated with unfavorable outcome [OR* *=* *4.159, 95% CI = (1.072, 16.137), *p *=* *0.039].

**Table 1:** The correlation between severity of MCA stenosis and the prevalence of NVF

Severity of stenosis	NVF *n* (%)	*r*	*p* value
50%~70%	0/14 (0)	0.467	<0.001
70%~99%	6/38 (15.8%)		
Occlusion	22/41 (53.7%)		

NVF: new vessels formation.

**Table 2:** The comparison of demographic and clinical data between patients with or without NVF

	With NVF (*n* = 28)	Without NVF (*n* = 65)	*p* value
Age, years	37.3 ± 6.0	38.1 ± 5.3	0.504
Male (%)	24 (85.7%)	54 (83.1%)	1.000
Hypertension (%)	8 (28.6%)	22/41 (53.7%)	0.634
Diabetes (%)	4 (14.3%)	5 (7.7%)	0.445
Hyperlipidemia (%)	0	2 (3.1%)	0.874
Smoking (%)	21 (75.0)	36 (55.4)	0.075
Antihypertensive drugs	5 (17.9%)	19 (29.2%)	0.250
Laboratory data			
Leukocyte count, 109/L	7.750 ± 2.608	7.179 ± 2.161	0.230
Fasting plasma glucose, mmol/L	5.194 ± 1.371	5.311 ± 1.418	0.890
LDL, mmol/L	2.0 (1.6, 2.7)	2.4 (2.0, 3.0)	0.063
HDL, mmol/L	1.005 ± 0.219	1.003 ± 0.257	0.900
TG, mmol/L	1.598 ± 1.075	1.619 ± 0.915	0.867
NIHSS score	3 (1,8)	2 (0, 7)	0.473
3 months unfavorable outcome (%)	6 (21.4%)	4 (6.2%)	0.061
Stroke recurrence (%)	5 (18.5%)	7 (10.9%)	0.330
Poor status of primary and secondary collateral circulation (%)	14 (50.0%)	19 (29.2%)	0.055

NVF: new vessels formation; LDL: low‐density lipoprotein cholesterin; HDL: high‐density lipoprotein; TG: triglyceride.

## 4 DISCUSSION

NVF is a common phenomenon observed through DSA examination in patients with isolated MCA steno‐occlusive disease (Hayashi, Suyama, & Nagata, 2010). The present study also found that NVF was significantly correlated with the severity of MCA stenosis in young ischemic stroke patients. In terms of stroke outcome, NVF independently predicts unfavorable outcome at 3 months after symptom onset. While, our results showed that NVF had no predictive value on symptom recurrence.

Brain collateral circulation can be classified into 3 categories: primary pathways (the circle of Willis), secondary pathways (leptomeningeal pathways of intracranial arteries), and new vessels formation through the processes of angiogenesis and arteriogenesis (Sheth & Liebeskind, 2015). Angiogenesis or arteriogenesis occurs when it is insufficient of primary and secondary collateral circulation (Liebeskind, 2003). Collateral circulation not only compensates for hypoperfusion, but also plays an important role in the clearance of necrotic fragments and micro‐emboli in the ischemic area (Manoonkitiwongsa, Jackson‐Friedman, McMillan, Schultz, & Lyden, 2001). The presence of NVF may represent a status of hypoperfusion and poor collaterals status in patients with steno‐occlusive MCA. Previous studies have proved that collateral circulation status greatly affects the initial symptom severity, infarct volume, and ultimate functional outcome (Liu et al., 2016; Sheth & Liebeskind, 2015). Our results showed a trend that NVF was more prevalent in patients with poor collateral circulations; thus, we supposed that may be the underlying reason for the difference of outcome between the 2 groups of patients. Xu et al. had found an unique new vessels formation phenomenon named deep tiny flow voids (DTFV) on MRI, which was defined as vascular formation along the steno‐occlusive MCA and distinct from moyamoya collaterals (Xu, Li, Gao, Hou, & Sun, 2016; Xu et al., 2014).Their studies indicated that these new vessels were in response to chronic cerebral ischemia.

The underlying pathophysiological mechanism of NVF is intriguing. It is unclear that NVF originates from the maturation of pre‐existing vessels (angiogenesis) or creation of new arteries (arteriogenesis) (Sheth & Liebeskind, 2015). Angiogenesis are induced by multiple cytokines released in the setting of ischemia (Rafat, Beck, Pena‐Tapia, Schmiedek, & Vajkoczy, 2009; Wittko‐Schneider, Schneider, & Plate, 2014; Zhang et al., 2000), while arteriogenesis are supposed to associate with increasing shear stress caused by artery stenosis (Troidl & Schaper, 2012; Van Royen et al., 2001). The retrospective design made it impossible to evaluate the cytokines level or vessel wall sheer stress of the patients. Further studies on the origin of NVF are needed.

The finding of NVF may have an impact on the choice of treatment strategy. Our study showed that NVF was more common in patients with poor collateral status, and it was associated with unfavorable clinical outcome. Thus, NVF could be applied to choose subgroups of patients with MCA steno‐occlusion whom may benefit from intensive therapy or interventional treatment. Thus, NVF might be a valuable predictor of clinical outcome for young ischemic stroke patients with MCA steno‐occlusion and helping to making treatment decision.

Our study has several limitations. First, we did not study the origin and development of NVF; it is unclear whether NVF occur before or after an ischemic event. Second, due to the small number of unfavorable group, we only performed a univariate logistic regression to analyze the association between NVF and stroke outcome. Finally, the retrospective design made it impossible to eliminate the effect of rehabilitation therapeutic differences on clinical outcome.

## CONFLICTS OF INTEREST

The authors declare that there are no conflicts of interest regarding the publication of this article.
